# Social Inequities in Cardiovascular Disease Risk Factors at Multiple Levels Persist Among Mothers in Texas

**DOI:** 10.3390/ijerph22030404

**Published:** 2025-03-10

**Authors:** Catherine Cubbin, Quynh Nhu (Natasha) B. La Frinere-Sandoval, Elizabeth M. Widen

**Affiliations:** 1 Steve Hicks School of Social Work, The University of Texas at Austin, 405 W. 25th Street, Austin, TX 78705, USA; natasha.bls@utexas.edu; 2 Department of Nutritional Sciences, College of Natural Sciences, The University of Texas at Austin, 200 W. 24th Street, Austin, TX 78712, USA; elizabeth.widen@austin.utexas.edu

**Keywords:** women’s health, cardiovascular disease, risk factors, social inequalities, neighborhoods, diabetes, hypertension, obesity, smoking

## Abstract

The life stage between the ages of 30–45 years for women is critical, given the competing demands of occupational advancement, intimate partner relationships, and childcare responsibilities. Cardiovascular disease (CVD) is the leading cause of death among women in the US, which is experienced inequitably by race/ethnicity/nativity and socioeconomic status and is embedded within geographic contexts. The objective of the current study was to examine social inequities in pre-pregnancy risk factors for cardiovascular disease. We analyzed 16 years of geocoded natality data in Texas (N = 2,089,588 births between 2005 and 2020 to mothers aged 30–45 years) linked with census tract- and county-level data. Dependent variables included pre-pregnancy diabetes, hypertension, obesity, and smoking. Independent variables included individual-level race/ethnicity/nativity and educational attainment, tract-level poverty and racial/ethnic concentrations, and county-level urban/rural status, with controls for other sociodemographic characteristics and time trend. Two-level, random intercept hierarchical generalized logistic models were used to estimate associations and model fit. Significant social inequities at the individual-, tract-, and county-levels in each risk factor were found. For example, tract-level variables had substantial and significant association with the four CVD risk factors, ranging from 13% to 72% higher odds in adjusted models. For all four risk factors, the more rural the county of residence was, the higher the odds of having the risk factor (24% to 256% higher odds). Individual-level social inequalities by race/ethnicity/nativity (ORs ranging from 0.04 to 2.12) and education (ORs ranging from 1.25 to 5.20) were also observed. Enhancing our understanding of this important period of life may enable policy and interventions to better support women through this critical life stage.

## 1. Introduction

The developmental life stage between the ages of 30–45 years, referred to as “Established Adulthood” [1] has been argued to be a critical yet understudied period in one’s life course, given the competing demands of occupational advancement, intimate partner relationships, and childcare responsibilities, the latter still falling much more heavily on mothers compared with fathers. Furthermore, race/ethnicity/nativity and educational attainment are likely to greatly influence the experience of established adulthood [1], with some social groups experiencing more stressors than others based on their (oftentimes intersecting) identities. Enhancing our understanding of this important period of life may enable policy and interventions to better support women who are going through this critical life stage, which has been termed the “rush hour of life” [1].

Cardiovascular disease (CVD) is the leading cause of death among women in the US [2], with diabetes, hypertension, obesity, and smoking among its most important proximal risk factors [3]. In 2021, a quarter of all deaths among women in the US were caused by heart disease or stroke [4], and a rather alarming recent trend found that the annual incidence of hospitalizations due to heart attacks increased for women within the relatively young stage of established adulthood [5]. Specifically, between 1995 and 2014, the proportion of women admitted with a heart attack who were aged 35–54 years compared with all those aged 35–74 increased from 21% to 31%. Importantly, the burden of CVD and its risk factors among women is inequitable by race/ethnicity/nativity and educational attainment: Black women have greater rates of cardiovascular risk factors (e.g., hypertension, obesity), events, and mortality than White women [6,7,8,9,10,11,12,13,14,15] and Black and Hispanic groups aged 20 and over had the highest rates of obesity [16]. Elevated risk of CVD is also present in several Asian subgroups, including South Asians and Filipinos [17,18]. Moreover, recent studies have shown that, compared to US-born adults, foreign-born adult residents have a greater likelihood of having chronic health conditions such as hypertension, diabetes, and reporting poor or fair instead of good health [19]. Foreign-born residents often have a greater lack of awareness about the risk factors for CVD, may experience more barriers to accessing health care [20,21,22]—including preventative health care--and, as such, may be less predisposed to seeking treatment or adopting positive behavior modifications to limit their risk [23]. Lower levels of educational attainment are also associated with a higher risk of impaired cardiovascular health overall and among foreign-born adults [23,24].

It is well established that CVD and its proximate risk factors are embedded within social and economic contexts, often operationalized into geographic levels, such as states, counties, or neighborhoods [25,26]. Neighborhood environments have been conceptualized as being health-damaging or health-promoting places, resulting from a long history of residential segregation. Consequently, these determinants of health have been theorized and investigated as mechanisms to explain racial/ethnic disparities in health overall, and in CVD and its risk factors specifically [25,26]. Populations of color and those with low socioeconomic status have a higher likelihood than Whites and those with higher socioeconomic status of residing in economically disadvantaged and segregated neighborhoods, which often lack opportunities to maintain a healthy lifestyle. These neighborhoods, while at the same time having considerable strengths, are often plagued by a variety of social issues that lead to CVD prevalence including high poverty rates, limited accessible exercise outlets, unhealthy food choices and advertising, lack of educational opportunities and high-quality health care, and public safety concerns [27]. Moreover, associations exist between cardiovascular health and favorable factors in the neighborhood environment, such as healthy food options, availability of exercise locations and walkability, healthy behavioral norms, as well as high neighborhood socioeconomic status [26].

Previous population-based studies found that neighborhood disadvantage (e.g., poverty rates or a socioeconomic deprivation index, at the block group-, tract- or ZIP code-level) is associated with cardiovascular health among women, including cardiovascular health/events [28,29,30], obesity [31,32,33,34,35], diabetes-related outcomes [36], blood pressure [37], and smoking [38]. However, this prior literature mostly focused on Black and White women and adult women of all ages, with less attention given to Asian and Hispanic women or immigrants.

Aging is happening more quickly in rural compared with urban areas, and urban-rural health disparities are increasing [12]. Living in rural areas may cause additional challenges for mothers in the Established Adulthood age group for a variety of reasons, including limited occupational opportunities, childcare, and health care, and longer distances to travel (thus, more compressed leisure time to spend on one’s own health). Recent trends showed that disparities in cardiovascular health outcomes, the leading cause of death among women, not only persist but have worsened for women living in rural versus urban communities [39]. Specifically, in the past decade, a doubling in the prevalence of pre-pregnancy hypertension has occurred among young rural women [40]. Other risk factors for CVD such as diabetes, tobacco use, and obesity remain elevated in rural women versus their urban counterparts [41].

The social-ecological model and ecosocial theory guide this study as well as a life course perspective [42,43,44,45]. The social-ecological model and ecosocial theory both emphasize the importance of structural and contextual factors as critical determinants of health outcomes, as well as multilevel influences on health. Ecosocial theory and the life course perspective both incorporate the idea of embodiment, e.g., accumulation of risk over time and how social factors “get into the body” and influence health outcomes during a specific life stage, in our case, Established Adulthood. The objective of the current study is to examine the effects of (1) individual-level race/ethnicity/nativity and socioeconomic status, (2) neighborhood-level socioeconomic and racial/ethnic characteristics, and (3) county-level urban/rural status on individual-level pre-pregnancy risk factors for CVD among persons who gave birth in Texas during the Established Adulthood life stage. To accomplish this objective, we analyzed 16 years of geocoded natality data in Texas (over 2 million births) linked with census tract- and county-level data. Texas is an ideal setting for the current study, with 254 counties (50% rural), 5265 census tracts (i.e., neighborhoods), and a racially/ethnically/nativity diverse population. Our study advances our knowledge on multiple contexts for multiple important CVD risk factors during an understudied critical period of women’s lives.

## 2. Materials and Methods

### 2.1. Data

We obtained individual-level birth certificate data https://www.cdc.gov/nchs/data/dvs/birth11-03final-ACC.pdf, accessed 20 February 2025) for all births from 2005 to 2020 in Texas (N = 6,368,228). We first filtered the data by age, excluding mothers under 30 years (63.4% of the total raw dataset) and over 45 years (0.1% of the total) for 2,325,386 births. We then excluded births that were missing data on any variable (10.1%, with missing geocodes and race/ethnicity being the largest contributors at 7.0% and 1.8%, respectively) or were in the “non-Hispanic Other” race category, resulting in a final sample size of 2,089,588 births to mothers aged 30–45.

Census tract-level (i.e., neighborhood-level) data came from the American Community Survey (ACS) from the U.S. Census. Five years of the ACS are pooled for tract-level estimates (e.g., the 2010 ACS is pooled from 2006–2010). Each birth certificate year was merged with the tract level data via FIPS codes corresponding to the ACS dataset release as follows: 2005–2009 birth certificates were merged with the 2010 ACS; 2010–2014 birth certificates were merged with the 2015 ACS; 2015–2019 birth certificates were merged with the 2019 ACS; and 2020 birth certificates were merged with the 2020 ACS.

**Figure 1:**
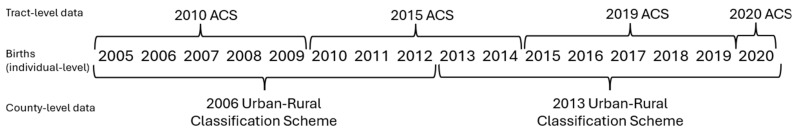
Schematic of data sources.

County level data came from the NCHS Urban-Rural Classification Scheme for Counties (https://www.cdc.gov/nchs/data-analysis-tools/urban-rural.html, accessed 20 February 2025). The 2005–2012 birth certificates were merged via FIPS codes with the 2006 version and the 2013–2020 birth certificates were merged with the 2013 version. Figure 1 presents a schematic of the tract- and county-level data sources merged with the birth records.

### 2.2. Variables

Individual-level dependent variables included prevalence of diabetes, hypertension, obesity, and smoking, all binary variables referring to pre-pregnancy. Diabetes is indicated by a check box for “Diagnosis prior to this pregnancy”. Hypertension is indicated by a check box for “Chronic”. Obesity is based on height in feet/inches and weight in pounds. First, we filtered out implausible heights by removing those below 4 feet. To calculate BMI based on the imperial system, mother’s weight in pounds was divided by the square of their height in inches and then multiplied by the constant 703. Smoking is based on whether the number of cigarettes or packs smoked was greater than zero for the three months before pregnancy. Individual-level covariates included age, race/ethnicity (non-Hispanic Asian/Pacific Islander, non-Hispanic Black, U.S.-born Hispanic, immigrant Hispanic, non-Hispanic White), marital status (not married, married), and educational attainment (did not complete high school, high school graduate/GED, some college, college graduate). Non-Hispanic White, married, and college graduate were selected as their respective reference groups since those groups have the most socioeconomic advantage and thus typically experience better health outcomes.

Tract level variables initially included percent poor, Black or African American (hereafter, “Black”), Hispanic or Latino (hereafter, “Hispanic”), or White. However, the Pearson correlation between percent Hispanic or Latino and percent White was −0.79; thus, we decided not to include percent White for brevity. Correlations among the other tract variables were between 0.19 and −0.54. For each ACS dataset, tract level variables were operationalized as z-scores with a mean of 0 and a standard deviation (SD) of 1 before merging with the birth certificate data. Continuous and squared terms for tract level variables were used in models to account for nonlinear associations.

The county-level variable was urban/rural status, categorized into three categories: (1) large metropolitan (MSA population of one million or more); (2) medium (MSA population of 250,000–999,999) or small (MSA population of less than 250,000) metropolitan, and (3) nonmetropolitan.

### 2.3. Analysis

For descriptive analyses, we calculated frequency distributions for all variables and prevalences for each dependent variable according to individual-, tract-, and county-level variables. Pseudo intraclass correlations coefficients (based on the formula τ00/(τ00 + π^3^/3)) were negligible, i.e., only 0.01–0.05, for between-county variance; however, they were more substantial, i.e., 0.10–0.24, for between-tract variance, thus justifying the use of two-level hierarchical generalized logistic models. For each dependent variable, we estimated a model with only the tract-level variable to determine the unadjusted neighborhood effect and then a model that included fixed effects for the individual-level variables, the county-level urban/rural status variable, and time period. The two-level random intercept model equations are below, using diabetes and percent poor as an example:

Level 1: Diabetesij = logistic [β0j + β1j(age)ij + β2j(Asian/Pacific Islander)ij + β3j(Black)ij + β4j(Hispanic immigrant)ij + β5j(Hispanic US-born)ij + β6j(unmarried)ij + β7j(did not complete high school)ij + β8j(high school/GED)ij + β9j(some college)ij + β10j(years 2005–2009)ij + β11j(years 2010–2014)ij + β12j(medium/small metropolitan)ij + β13j(nonmetropolitan)ij]

Level 2: β0j = ɣ00 + ɣ01(%poor)j + ɣ02(%poor^2^)j + µ0j

β1j… β13j = ɣ10… ɣ130

All analyses were conducted in SAS 9.4. The GLIMMIX procedure was used with Laplace estimation and model fit was determined with log-likelihood ratio tests, AIC, and BIC.

## 3. Results

Table 1 presents descriptive statistics for the population. Not surprisingly, most people were in their early thirties. Over 80% of the population identified as Hispanic or White and nearly that many were married at the time of birth. Almost half of the population had a college degree while 16% did not complete high school. A similar proportion of the population lived in either low (15%) or high (13%) poverty tracts. Notably, there were no tracts that were considered low for % Black (i.e., less than 1 SD below the mean), while 11% of the population lived in high % Black tracts; in other words, nearly 90% of the population lived in Black concentration tracts that were within 1 SD of the mean. Similar to poverty, 11% lived in low Hispanic concentration tracts and 19% lived in high Hispanic concentration tracts. Three quarters of people giving birth in this age range lived in large metropolitan counties while only 6% lived in nonmetropolitan counties.

As expected with this relatively young population, risk factor prevalences were low overall except for obesity, where over a quarter of the population were classified with obesity. Prevalences of diabetes, hypertension and obesity increased with age while smoking rates decreased with age. Asian/Pacific Islanders generally had the most favorable risk factor profiles and Black and U.S.-born Hispanic persons generally had the least favorable profiles. Immigrant Hispanic and White persons were in between except that immigrant Hispanics had a higher prevalence of diabetes and Whites had the highest smoking prevalence of any racial/ethnic group. Married people had lower risk factor levels than unmarried people, especially for smoking which was over three times higher for unmarried (6.5%) compared with married (2.0%) people. Diabetes prevalence went down uniformly with increasing education. However, for hypertension, obesity, and smoking, prevalences were highest for high school graduates and those with some college education, lowest for college graduates, and in between for those who did not complete high school. Except for smoking, risk factor prevalence increased with increasing poverty concentrations at the tract level. Risk factor prevalences were highest in tracts with the highest concentration of Black persons as well as the highest concentration of Hispanic persons (except for hypertension and smoking). Finally, prevalence rates for all risk factors increased with rurality. For example, obesity prevalence increased from 24% to 35% and smoking increased from 2% to 8% in large metropolitan vs. nonmetropolitan counties, respectively.

Results from the diabetes models are presented in Table 2. Each SD increase in poverty concentration was associated with a 90% higher likelihood of diabetes, which leveled off at higher levels of poverty as indicated by the lower likelihood for the squared term (OR = 0.68); these effects remained (ORs 1.39 and 0.80) in the model adjusted for individual-level characteristics, urban/rural status, and time period. In contrast, % Black was not associated with diabetes in either model. However, % Hispanic followed a very similar pattern as poverty concentration. Each year of age was associated with a 9% higher odds of diabetes and compared with White persons, every other racial/ethnic group had higher odds of diabetes, especially U.S.-born Hispanic persons with a twofold higher odds of diabetes. Educational attainment followed a gradient pattern, with odds increasing at lower levels compared with college graduates. Nonmetropolitan area residence was associated with higher odds of diabetes compared with large metropolitan area residence and the time trend indicated lower odds in the earliest vs. latest time period, reflecting increasing diabetes prevalence over time.

Each neighborhood-level variable was associated with hypertension, in both unadjusted and adjusted models (Table 3). Each standard deviation increase was associated with 13–57% higher odds, depending on the tract indicator, with increases leveling off at higher levels. Every other variable was also associated with risk of hypertension, with older age, Black race/ethnicity, not being married, lower education, and less urbanized residence associated with higher odds compared with their respective reference groups. In contract, Asian/Pacific Islanders and Hispanic immigrants had lower odds of hypertension than White, while U.S.-born Hispanics had about the same or slightly higher odds. Persons who gave birth in the earlier time periods had lower odds of hypertension compared to those who gave birth in the latest time period.

Table 4 presents the models for obesity and similar effects were found for each tract variable. Each SD increase in poverty, Black, or Hispanic concentration was associated with a 12–33% higher odds of obesity, and those effects became weaker at higher tract concentrations for each (adjusted models). Not surprisingly, older age was associated with higher odds of obesity. Compared with White persons, Asian/Pacific Islanders and Hispanic immigrants had lower odds of obesity, while Black and U.S.-born Hispanic persons had higher odds. Unmarried persons had slightly higher odds of obesity than married persons, and persons at all levels of educational attainment with less than a college degree had higher odds of obesity compared with college graduates. Urban/rural status followed a gradient, with nonmetropolitan counties having the highest odds of obesity, followed by medium or small metropolitan counties, compared with large metropolitan counties. The time trend indicates that the odds of obesity increased over time as expected.

Like obesity, each tract variable had a similar association with smoking (Table 5). Each SD increase in poverty, Black, or Hispanic concentration was associated with a 13–72% higher odds of smoking, and those effects became weaker at higher tract concentrations for each (adjusted models). Older age was associated with lower odds of smoking, and all racial/ethnic groups had much lower odds of smoking compared with White persons, especially Hispanic immigrants (OR = 0.04). Unmarried persons had about three times higher odds of obesity than married persons, and educational attainment followed an inverse gradient pattern; strikingly, those without a high school degree had five times higher odds of smoking compared with college graduates. Urban/rural status followed a gradient, with nonmetropolitan counties having the highest odds (about 2.5 times higher), followed by medium or small metropolitan counties (about 1.7–2 times higher), compared with large metropolitan counties. The time trend indicates that the odds of smoking decreased over time as expected.

For each dependent variable, fit statistics indicated that adjusted models were better fitting models than unadjusted models and proportion of between-tract variance explained was substantial, ranging from 38–41% for hypertension, 44–51% for diabetes, 50–56% for obesity, and 73–76% for smoking, depending on the neighborhood variable modeled.

## 4. Discussion

We found that the economic and racial/ethnic context of neighborhoods had an impact on important CVD risk factors for pregnant persons experiencing the “rush hour” life stage. With one exception (% Black and risk of diabetes), each tract-level variable had a substantial and signification association with the four CVD risk factors, ranging from 13% to 72% higher odds after adjustment for demographic and socioeconomic factors at the individual level, urban/rural status, and time. In addition, we found that the neighborhood-level variable associations were nonlinear; i.e., associations became weaker at higher tract-level concentrations (evidenced by lower odds for the tract variable squared terms). Furthermore, for all four risk factors, the more rural the county of residence was, the higher the odds of having the risk factor. This was especially the case for smoking, with persons having about two and a half higher odds of being a current smoker if they lived in a nonmetropolitan county vs. a large metropolitan county in adjusted models. Expected and well documented demographic and socioeconomic disparities were also observed in our study. Finally, time trends were also found, with lower odds in the earlier periods for most risk factors (diabetes, hypertension, obesity), but higher odds in the earlier periods for current smoking, reflecting general patterns in prevalence rates over time for these behaviors and outcomes.

Our findings on diabetes are in line with a previous study that found that living in socioeconomically disadvantaged neighborhoods was associated with metabolic syndrome among non-diabetic Black women aged 21 and over in the Jackson Heart Study [36]. The same study also found that perceived neighborhood safety was associated with elevated glucose and weight circumference [36], suggesting that perhaps neighborhood safety is an explanatory mediator of the neighborhood socioeconomic status-diabetes relationship. Schiff et al. [37] conducted a longitudinal analysis of women aged 42–52 at baseline from seven diverse clinical sites and found that women living in the most socioeconomically “vulnerable” neighborhoods had the largest increase in systolic blood pressure over time, providing evidence that neighborhood context is causally related to hypertension, a claim that we cannot make with our cross-sectional study design, despite findings that are in alignment. Our findings on obesity are also in line with previous studies [31,32,33,34,35]. Two studies in particular were most closely aligned with our study in terms of data source or age group: Mendez et al. [33] also analyzed birth records (one county in Pennsylvania) and pre-pregnancy obesity and found that high neighborhood poverty was associated with obesity for both Black and White women of childbearing age; and Sheehan et al. [35] analyzed mothers in California aged 20–57 years, and found that living in a neighborhood that experienced long-term poverty was associated with higher odds of obesity. Neighborhood socioeconomic disadvantage & affluence was associated with daily smoking in a random sample of adults aged 18 and over (with no differences identified in the associations by gender) [38], which also aligns with our findings despite the different operationalization of smoking between the studies.

Our findings on urban/rural status are in line with most other existing research on preconception health. Two studies using U.S. birth records found that pre-pregnancy hypertension rates [40] in 2007–2018 and diabetes [46] rates in 2011–2019 were higher in rural compared with urban counties. Similarly, a report using Tennessee PRAMS data from 2020 found that smoking rates, but not obesity rates, were higher in rural compared with urban counties [47]. In contrast, a study of birth records from 2004–2006 in South Carolina did find that obesity rates were higher in rural compared with urban counties [48]. All of these studies included childbearing women of all ages, however, in contrast to our limited age range.

Given the young age of persons in this study, (about 95% were in their thirties) and that for nearly everyone these risk factors are entirely preventable, our findings on racial/ethnic, socioeconomic, neighborhood context, and urban/rural disparities point to structural conditions as targets for intervention. By design, all persons in our study were parents, sometimes for the first time but, in most cases, they were already parenting other children. In addition to childcare responsibilities, the 30s are years in which most people are working and navigating intimate partner relationships. Some may even be caring for aging parents. These multiple roles are stressful even in situations with supportive and adequate social and economic resources [49], making it difficult to prioritize one’s health through behaviors and preventative care. Belonging to a marginalized group (person of color, immigrant, someone with low educational attainment or income) and/or residing in a community context that presents barriers to making healthy choices [50,51] may compound the “regular” stressors that come with having multiple roles. These compounding stressors may be related to discrimination, financial strain, safety, access to healthy, affordable food and/or places for physical activity, among others [52]. Our study contributes further to the body of work on neighborhood and community context as targets for policy and place-based intervention.

The strengths of our study are its large population size, allowing us to focus on women during a narrowly defined Established Adulthood phase. The large population also allowed us to distinguish Hispanic persons according to nativity. Our data also had a high degree of geocoding accuracy with nearly all records geocoding to the street level. However, relying on birth certificate data has its limitations. First, pre-pregnancy diabetes and hypertension are based on a diagnosis and persons who do not seek heath care may be unaware of their condition. Diabetes has also been found to be underreported on birth certificate data compared with hospital records [53]. Smoking is likely to be underreported because of social desirability bias and pre-pregnancy weight status has been found to be subject to measurement error [54] compared with hospital records. The current smoker definition was also based on having smoked at least one cigarette during the three months prior to becoming pregnant, which would include both heavy and light smokers and may not reflect usual smoking patterns/risk if women were trying to smoke less or quit if they were trying to become pregnant. The smoking item also does not specify whether the product was tobacco or whether cigarettes included both electronic and traditional ones. Furthermore, although we refer to women throughout the study, we are aware than not all birthing people identify as women. We did not disaggregate racial/ethnic groups beyond Asian/Pacific Islander, Black, Hispanic, or White. Also, many women move during pregnancy and thus their tract/county at the time of birth may be different from when they became pregnant, which may be the more important exposure time of interest to examine tract/county effects. Finally, we cannot make causal inferences with our observational, cross-sectional study design, and due to our study design our results may not be generalizable to non-childbearing women who may experience lower (or higher) levels of risk factors.

Future work should examine within-group effects since racial/ethnic and socioeconomic disparities may further exacerbate the neighborhood effects and urban/rural inequities we observed. Hypothetically, groups who have been impacted by structural racism and institutional discrimination (women of color, immigrant women, women with low education or income) may be more impacted by their neighborhoods than their more socially advantaged counterparts and these impacts may be more pronounced in rural areas. In addition to neighborhoods, examining county level contexts is of great importance for reducing CVD since local governments at the county level are responsible for approving comprehensive plans for county’s growth as well as providing a wide range of social services to its residents. It is at this level that many important decisions are made that may significantly impact health, healthcare quality, and access [55]. Although it was beyond the scope of the current study, future studies should examine whether neighborhood and county environments have a differential impact by race/ethnicity or socioeconomic status, an area of work that we are currently investigating.

## 5. Conclusions

In conclusion, we found substantial and significant social inequities at the individual-, tract-, and county-levels for four important proximate risk factors for cardiovascular disease among women in the emerging adulthood life stage. Tract-level variables describing poverty and racial/ethnic concentrations had substantial and significant associations with the four CVD risk factors, ranging from 13% to 72% higher odds in adjusted models. For all four risk factors, the more rural the county of residence was, the higher the odds of having the risk factor (24% to 256% higher odds). Individual-level social inequalities by race/ethnicity/nativity (ORs ranging from 0.04 to 2.12) and education (ORs ranging from 1.25 to 5.20) were also observed. While the racial/ethnic disparities varied substantially across risk factors, the education disparities were consistent (lower education was associated with higher risk). Our study enhances our understanding of CVD risk that may enable policy and interventions to better support them going through this critical life stage. The solutions proposed by the American Heart Association and American Stroke Association [41] that include Medicaid expansion, investments in the rural workforce, and economic development in rural areas are important steps to reduce urban/rural disparities in CVD risk, along with place-based built environment changes across all communities to make it easier to live healthier lives and reduce disparities in cardiovascular disease.

## Figures and Tables

**Table 1:** Descriptive characteristics, birth records in Texas, 2005–2020, N = 2,089,588.

	Distribution		Risk Factor Prevalence ^1^			
	N	%	Diabetes	Hypertension	Obesity	Smoking
Overall	2,089,588	100.0%	1.1%	1.9%	25.9%	3.0%
**Individual-level**						
Age						
30–35 years	1,508,945	72.2%	1.0%	1.5%	25.3%	3.1%
36–40 years	497,650	23.8%	1.6%	2.5%	27.3%	2.6%
41–45 years	82,993	4.0%	2.2%	3.8%	28.2%	2.4%
Race/Ethnicity						
Asian/Pacific Islander	172,379	8.2%	0.9%	0.8%	7.5%	0.5%
Black	214,449	10.3%	1.3%	4.2%	37.2%	3.8%
Hispanic, U.S.-born	381,628	18.3%	1.8%	2.2%	39.5%	2.5%
Hispanic, immigrant	486,085	23.3%	1.5%	1.3%	24.8%	0.5%
White	835,047	40.0%	0.7%	1.7%	21.2%	5.0%
Marital status						
Married	1,640,361	78.5%	1.0%	1.7%	24.0%	2.0%
Not married	449,227	21.5%	1.5%	2.5%	32.9%	6.5%
Educational attainment						
Did not complete high school	335,035	16.0%	1.9%	1.7%	30.0%	2.8%
High school graduate or GED	352,839	16.9%	1.6%	2.3%	31.5%	5.2%
Some college	368,679	17.6%	1.3%	2.4%	34.7%	5.6%
College graduate	1,033,035	49.4%	0.7%	1.5%	19.5%	1.4%
Tract-level * (SD = standard deviation)						
% Poor						
<1 SD from the mean	314,531	15.1%	0.7%	1.3%	16.5%	1.8%
Within 1 SD of the mean	1,496,999	71.6%	1.1%	1.9%	26.4%	3.3%
>1 SD from the mean	278,058	13.3%	1.8%	2.1%	33.9%	2.6%
% Black or African American						
<1 SD from the mean	0	0.0%	--	--	--	--
Within 1 SD of the mean	1,850,450	88.6%	1.1%	1.8%	25.2%	3.0%
>1 SD from the mean	239,138	11.4%	1.4%	2.7%	31.5%	3.2%
% Hispanic or Latino						
<1 SD from the mean	238,032	11.4%	0.7%	1.5%	18.2%	3.5%
Within 1 SD of the mean	1,457,237	69.7%	1.1%	1.9%	25.0%	3.3%
>1 SD from the mean	394,319	18.9%	1.7%	1.8%	34.0%	1.7%
**County-level urban/rural status**						
Large metropolitan	1,556,324	74.5%	1.1%	1.8%	23.6%	2.3%
Medium or small metropolitan	402,790	19.3%	1.2%	2.0%	31.6%	4.2%
Nonmetropolitan	130,474	6.2%	1.6%	2.8%	35.2%	7.5%

* standardization based on all tracts in Texas; **^1^** *p* values all < 0.001 based on chi-square tests.

**Table 2:** Logistic regression models (odds ratios and 95% confidence intervals) for diabetes, birth records in Texas, 2005–2020, ages 30–45, N = 2,089,588.

Fixed Effects	% Poor, Unadjusted	% Poor, Adjusted	% Black/African American, Unadjusted	% Black/African American, Adjusted	% Hispanic/ Latino, Unadjusted	% Hispanic/ Latino, Adjusted
Age	--	1.09 (1.09–1.10) *	--	1.09 (1.09–1.10) *	--	1.09 (1.09–1.10) *
Race/ethnicity	--		--		--	
Asian/Pacific Islander		1.44 (1.36–1.53) *		1.49 (1.41–1.58) *		1.47 (1.39–1.56) *
Black		1.62 (1.54–1.70) *		1.71 (1.62–1.80) *		1.68 (1.60–1.76) *
Hispanic, U.S.-born		1.98 (1.90–2.06) *		2.06 (1.98–2.15) *		1.91 (1.84–1.99) *
Hispanic, immigrant		1.33 (1.27–1.38) *		1.42 (1.36–1.49) *		1.31 (1.26–1.37) *
White		1.00		1.00		1.00
Marital status	--		--		--	
Married		1.00		1.00		1.00
Not married		1.00 (0.97–1.02)		1.01 (0.98–1.04)		1.00 (0.97–1.03)
Educational attainment	--		--		--	
<High school		1.90 (1.80–1.96) *		1.99 (1.91–2.08) *		1.91 (1.83–2.00) *
High school graduate/GED		1.69 (1.63–1.76) *		1.76 (1.69–1.83) *		1.72 (1.65–1.79) *
Some college		1.52 (1.47–1.58) *		1.56 (1.50–1.62) *		1.72 (1.65–1.79) *
College graduate		1.00		1.00		1.00
% Poor	1.90 (1.76–1.94) *	1.39 (1.33–1.46) *	--	--	--	--
% Poor ^2^	0.68 (0.65–0.72) *	0.80 (0.80–0.85) *				
% Black or African American	--	--	1.02 (1.00–1.08)	0.99 (0.95–1.04)	--	--
% Black or African American ^2^			1.06 (1.01–1.12) ^	1.04 (0.99–1.09)		
% Hispanic or Latino	--	--	--	--	2.02 (1.90–2.18) *	1.46 (1.36–1.58) *
% Hispanic or Latino ^2^					0.70 (0.61–0.71) *	0.80 (0.72–0.83) *
Urban/rural status	--		--		--	
Large metropolitan		1.00		1.00		1.00
Medium/small metropolitan		0.90 (0.87–0.94) *		0.99 (0.95–1.04)		0.97 (0.92–1.01)
Nonmetropolitan		1.24 (1.16–1.31) *		1.32 (1.24–1.40) *		1.35 (1.27–1.43) *
Time	--		--		--	
Years 2005–2009		0.85 (0.81–0.88) *		0.84 (0.81–0.87) *		0.84 (0.81–0.87) *
Years 2010–2014		0.97 (0.94–1.00)		0.97 (0.94–1.00)		0.97 (0.94–1.00) ^
Years 2015–2020		1.00		1.00		1.00
**Random effect**	0.25	0.19	0.38	0.22	0.27	0.20
**Fit statistics**						
−2 log likelihood	257,431	252,130	258,368	252,361	257,442	252,187
AIC	257,439	252,164	258,376	252,395	257,450	252,221
BIC	257,467	252,285	258,404	252,516	257,479	252,342

^ *p* < 0.05; * *p* < 0.001; ^2^ refers to squared terms.

**Table 3:** Logistic regression models (odds ratios and 95% confidence intervals) for hypertension, birth records in Texas, 2005–2020, ages 30–45, N = 2,089,588.

Fixed Effects	% Poor, Unadjusted	% Poor, Adjusted	% Black/African American, Unadjusted	% Black/African American, Adjusted	% Hispanic/ Latino, Unadjusted	% Hispanic/ Latino, Adjusted
Age	--	1.11 (1.10–1.11) *	--	1.11 (1.10–1.11) *	--	1.11 (1.10–1.11) *
Race/ethnicity	--		--		--	
Asian/Pacific Islander		0.51 (0.49–0.54) *		0.52 (0.49–0.55) *		0.52 (0.49–0.55) *
Black		2.11 (2.04–2.17) *		2.09 (2.02–2.16) *		2.12 (2.05–2.18) *
Hispanic, U.S.-born		1.03 (1.00–1.06)		1.08 (1.04–1.11) *		1.03 (1.00–1.06)
Hispanic, immigrant		0.57 (0.55–0.59) *		0.60 (0.58–0.62) *		0.58 (0.56–0.60) *
White		1.00		1.00		1.00
Marital status	--		--		--	
Married		1.00		1.00		1.00
Not married		1.15 (1.12–1.17) *		1.15 (1.13–1.18) *		1.16 (1.13–1.18) *
Educational attainment	--		--		--	
<High school		1.25 (1.20–1.30) *		1.26 (1.22–1.31) *		1.27 (1.22–1.31) *
High school graduate/GED		1.33 (1.29–1.37) *		1.35 (1.31–1.39) *		1.34 (1.30–1.38) *
Some college		1.32 (1.28–1.36) *		1.33 (1.29–1.37) *		1.32 (1.29–1.36) *
College graduate		1.00		1.00		1.00
% Poor	1.39 (1.34–1.46) *	1.29 (1.24–1.34) *	--	--	--	--
% Poor ^2^	0.78 (0.74–0.81) *	0.82 (0.79–0.85) *				
% Black or African American	--	--	1.20 (1.16–1.25) *	1.13 (1.09–1.17) *	--	--
% Black or African American ^2^			0.99 (0.96–1.03)	0.95 (0.91–0.98) +		
% Hispanic or Latino	--	--	--	--	1.57 (1.47–1.70) *	1.57 (1.48–1.67) *
% Hispanic or Latino ^2^					0.65 (0.61–0.69) *	0.67 (0.62–0.71) *
Urban/rural status	--		--		--	
Large metropolitan		1.00		1.00		1.00
Medium/small metropolitan		1.10 (1.06–1.14) *		1.18 (1.14–1.23) *		1.19 (1.14–1.23) *
Nonmetropolitan		1.49 (1.42–1.57) *		1.63 (1.55–1.71) *		1.63 (1.56–1.72) *
Time	--		--		--	
Years 2005–2009		0.74 (0.72–0.76) *		0.75 (0.72–0.77) *		0.74 (0.72–0.77) *
Years 2010–2014		0.88 (0.86–0.90) *		0.89 (0.87–0.91) *		0.89 (0.86–0.91) *
Years 2015–2020		1.00		1.00		1.00
**Random effect**	0.26	0.17	0.24	0.18	0.27	0.17
**Fit statistics**						
−2 log likelihood	382,566	370,654	382,409	370,731	382,694	370,606
AIC	382,574	370,688	382,417	370,765	382,702	370,640
BIC	382,603	370,809	382,445	370,886	382,730	370,760

+ *p* < 0.01; * *p* < 0.001; ^2^ refers to squared terms.

**Table 4:** Logistic regression models (odds ratios and 95% confidence intervals) for Obesity, birth records in Texas, 2005–2020, ages 30–45, N = 2,089,588.

Fixed Effects	% Poor, Unadjusted	% Poor, Adjusted	% Black/African American, Unadjusted	% Black/African American, Adjusted	% Hispanic/ Latino, Unadjusted	% Hispanic/ Latino, Adjusted
Age	--	1.03 (1.03–1.03) *	--	1.03 (1.03–1.03) *	--	1.03 (1.03–1.03) *
Race/ethnicity	--		--		--	
Asian/Pacific Islander		0.33 (0.33–0.34) *		0.33 (0.33–0.34) *		0.34 (0.33–0.34) *
Black		1.74 (1.72–1.76) *		1.73 (1.71–1.75) *		1.74 (1.72–1.76) *
Hispanic, U.S.-born		1.72 (1.70–1.74) *		1.73 (1.71–1.75) *		1.70 (1.68–1.71) *
Hispanic, immigrant		0.81 (0.80–0.82) *		0.81 (0.81–0.82) *		0.80 (0.79–0.81) *
White		1.00		1.00		1.00
Marital status	--		--		--	
Married		1.00		1.00		1.00
Not married		1.02 (1.01–1.03) *		1.02 (1.01–1.03) *		1.02 (1.01–1.03) *
Educational attainment	--		--		--	
<High school		1.41 (1.39–1.43) *		1.42 (1.40–1.43) *		1.41 (1.39–1.43) *
High school graduate/GED		1.36 (1.34–1.37) *		1.36 (1.35–1.37) *		1.36 (1.35–1.37) *
Some college		1.54 (1.53–1.56) *		1.54 (1.53–1.56) *		1.54 (1.53–1.56) *
College graduate		1.00		1.00		1.00
% Poor	1.30 (1.28–1.33) *	1.24 (1.22–1.26) *	--	--	--	--
% Poor ^2^	0.86 (0.84–0.87) *	0.88 (0.86–0.89) *				
% Black or African American	--	--	1.07 (1.04–1.09) *	1.13 (1.11–1.16) *	--	--
% Black or African American ^2^			1.02 (1.00–1.05)	0.95 (0.93–0.97) *		
% Hispanic or Latino	--	--	--	--	1.32 (1.27–1.37) *	1.34 (1.30–1.39) *
% Hispanic or Latino ^2^					0.94 (0.91–0.98) +	0.85 (0.82–0.88) *
Urban/rural status	--		--		--	
Large metropolitan		1.00		1.00		1.00
Medium/small metropolitan		1.28 (1.25–1.31) *		1.40 (1.37–1.44) *		1.29 (1.26–1.32) *
Nonmetropolitan		1.49 (1.45–1.54) *		1.61 (1.56–1.66) *		1.58 (1.54–1.63) *
Time	--		--		--	
Years 2005–2009		0.74 (0.73–0.75) *		0.74 (0.73–0.75) *		0.74 (0.73–0.75) *
Years 2010–2014		0.86 (0.85–0.86) *		0.86 (0.85–0.86) *		0.86 (0.85–0.86) *
Years 2015–2020		1.00		1.00		1.00
**Random effect**	0.29	0.16	0.35	0.18	0.29	0.16
**Fit statistics**						
−2 log likelihood	2,285,395	2,213,221	2,286,169	2,213,688	2,284,944	2,213,103
AIC	2,285,403	2,213,255	2,286,177	2,213,722	2,284,952	2,213,137
BIC	2,285,432	2,213,376	2,286,205	2,213,843	2,284,980	2,213,258

+ *p* < 0.01; * *p* < 0.001; ^2^ refers to squared terms.

**Table 5:** Logistic regression models (odds ratios and 95% confidence intervals) for smoking, birth records in Texas, 2005–2020, ages 30–45, N = 2,089,588.

Fixed Effects	% Poor, Unadjusted	% Poor, Adjusted	% Black/African American, Unadjusted	% Black/African American, Adjusted	% Hispanic/ Latino, Unadjusted	% Hispanic/ Latino, Adjusted
Age	--	0.99 (0.99–0.99) *	--	0.99 (0.99–0.99) *	--	0.99 (0.99–0.99) *
Race/ethnicity	--		--		--	
Asian/Pacific Islander		0.16 (0.15–0.17) *		0.16 (0.15–0.17) *		0.16 (0.15–0.17) *
Black		0.42 (0.41–0.44) *		0.41 (0.40–0.43) *		0.42 (0.41–0.43) *
Hispanic, U.S.-born		0.25 (0.25–0.26) *		0.25 (0.25–0.26) *		0.28 (0.27–0.28) *
Hispanic, immigrant		0.04 (0.04–0.04) *		0.04 (0.04–0.04) *		0.04 (0.04–0.04) *
White		1.00		1.00		1.00
Marital status	--		--		--	
Married		1.00		1.00		1.00
Not married		2.95 (2.89–3.00) *		2.94 (2.89–3.00) *		2.99 (2.94–3.05) *
Educational attainment	--		--		--	
<High school		4.99 (4.83–5.15) *		5.02 (4.87–5.19) *		5.20 (5.03–5.37) *
High school graduate/GED		3.84 (3.75–3.94) *		3.85 (3.75–3.95) *		3.94 (3.85–4.05) *
Some college		3.24 (3.16–3.32) *		3.28 (3.20–3.36) *		3.34 (3.26–3.42) *
College graduate		1.00		1.00		1.00
% Poor	1.19 (1.14–1.24) *	1.19 (1.14–1.23) *	--	--	--	--
% Poor ^2^	0.83 (0.79–0.87) *	0.83 (0.80–0.86) *				
% Black or African American	--	--	0.99 (0.94–1.04)	1.13 (1.09–1.18) *	--	--
% Black or African American ^2^			1.06 (1.01–1.11) ^	0.92 (0.89–0.96) *		
% Hispanic or Latino	--	--	--	--	1.25 (1.16–1.36) *	1.72 (1.62–1.83) *
% Hispanic or Latino ^2^					0.59 (0.54–0.64) *	0.47 (0.44–0.50) *
Urban/rural status	--		--		--	
Large metropolitan		1.00		1.00		1.00
Medium/small metropolitan		1.66 (1.59–1.73) *		1.73 (1.66–1.80) *		2.01 (1.93–2.09) *
Nonmetropolitan		2.35 (2.24–2.50) *		2.49 (2.38–2.61) *		2.56 (2.44–2.68) *
Time	--		--		--	
Years 2005–2009		1.43 (1.39–1.46) *		1.43 (1.40–1.46) *		1.44 (1.40–1.47) *
Years 2010–2014		1.29 (1.26–1.31) *		1.29 (1.26–1.31) *		1.29 (1.27–1.32) *
Years 2015–2020		1.00		1.00		1.00
**Random effect**	1.03	0.29	1.05	0.29	0.93	0.25
**Fit statistics**						
−2 log likelihood	525,279	458,737	525,330	458,744	524,716	457,971
AIC	525,287	458,771	525,338	458,778	524,724	458,005
BIC	525,315	458,892	525,367	458,899	524,752	458,125

^ *p* < 0.05; * *p* < 0.001; ^2^ refers to squared terms.

## Data Availability

Data are unavailable due to ethical restrictions.
